# Supplementary figures and images for: Stimulation of the catalytic activity of the tyrosine kinase Btk by the adaptor protein Grb2
Source: eLife. 2023 Apr 26;12:e82676. doi: 10.7554/eLife.82676 (PMC10132808; doi:10.7554/eLife.82676)

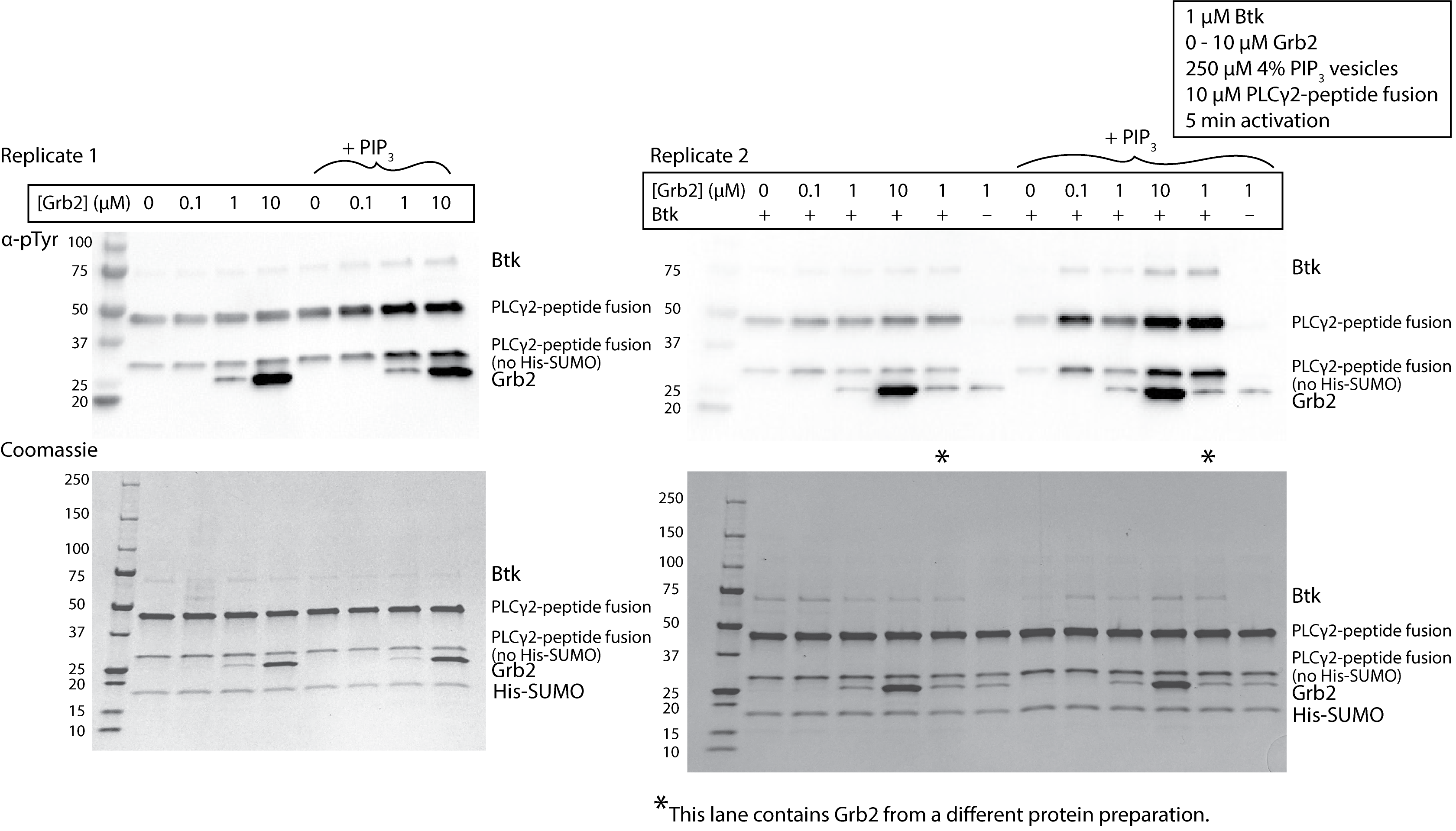

Supplement: Figure 3—source data 1. [file elife-82676-fig3-data1.zip › Figure 3 source data/Figure 3C-source data 5.png]

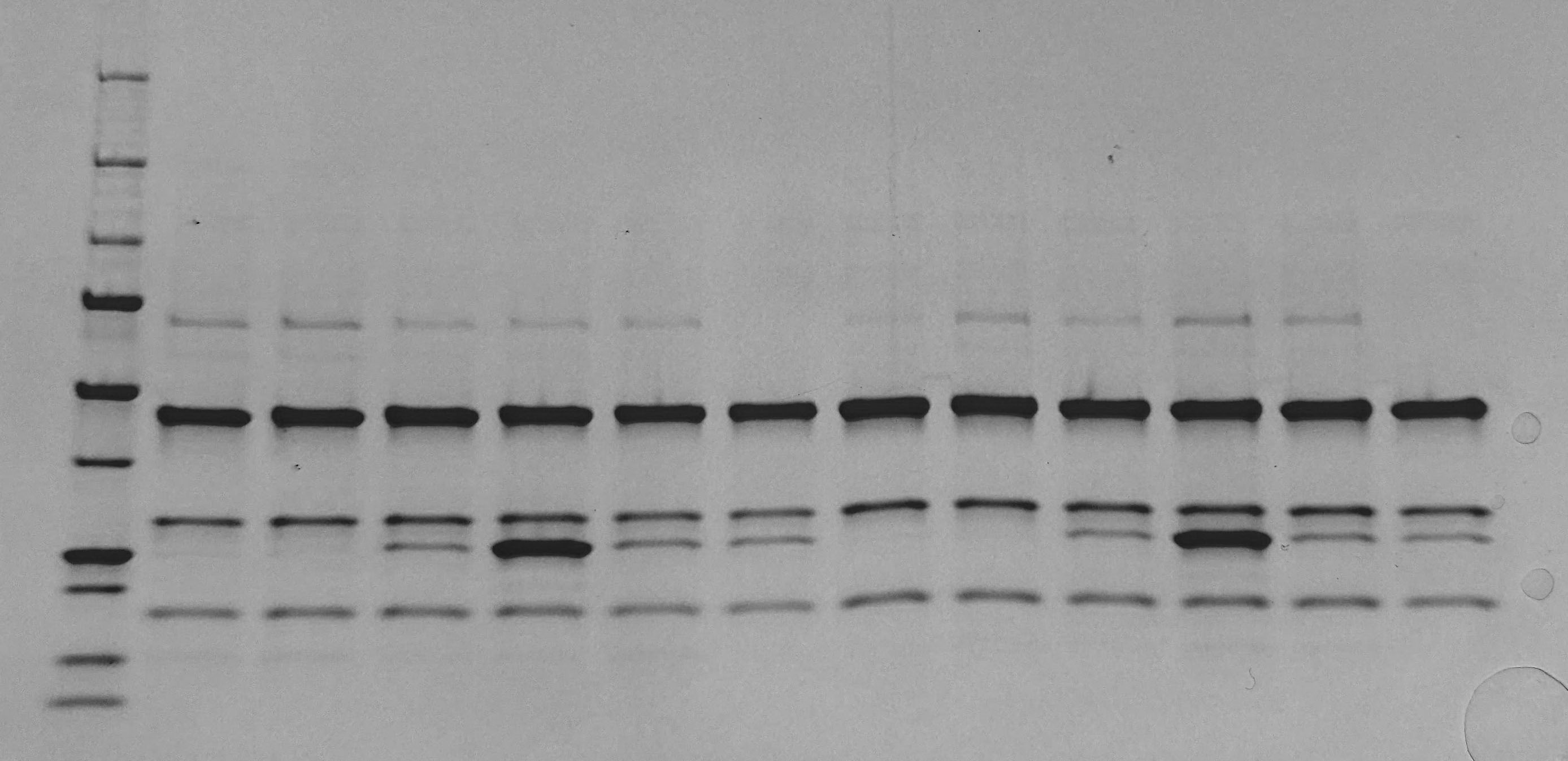

Supplement: Figure 3—source data 1. [file elife-82676-fig3-data1.zip › Figure 3 source data/Figure 3C-source data 4.jpg]

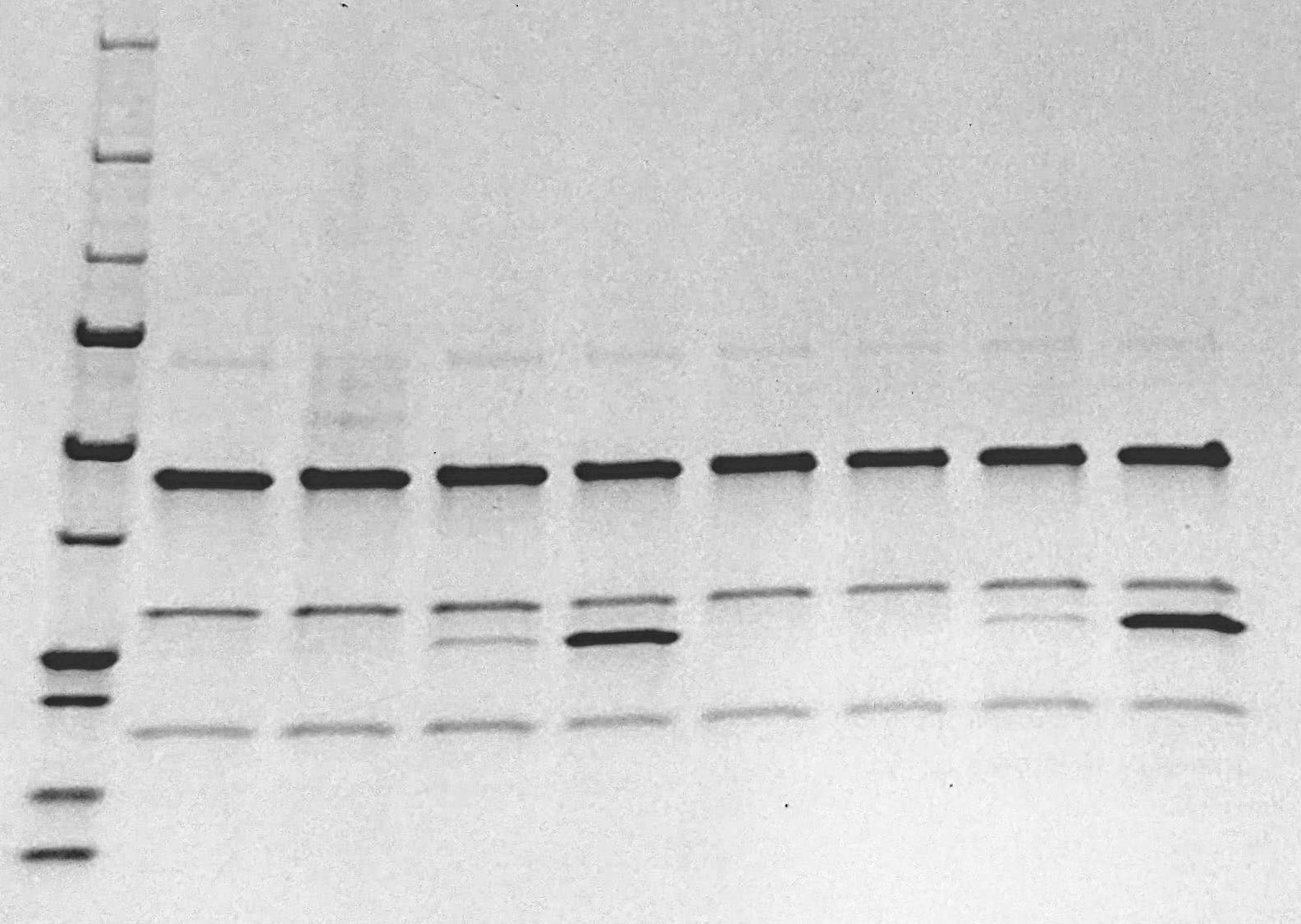

Supplement: Figure 3—source data 1. [file elife-82676-fig3-data1.zip › Figure 3 source data/Figure 3C-source data 2.jpg]

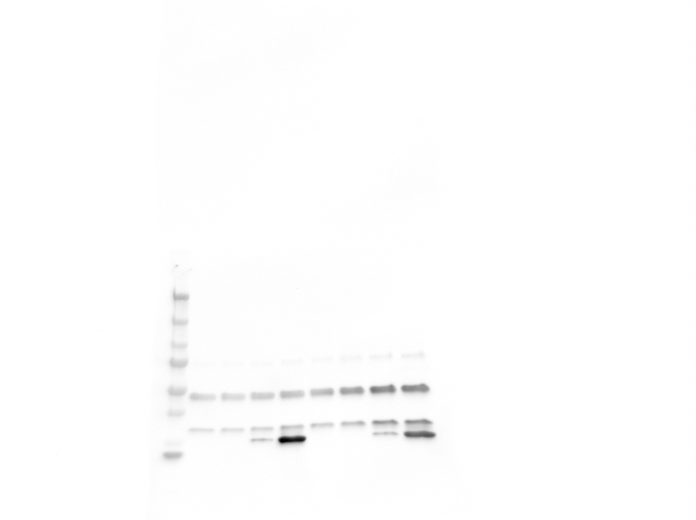

Supplement: Figure 3—source data 1. [file elife-82676-fig3-data1.zip › Figure 3 source data/Figure 3C-source data 3.tif]

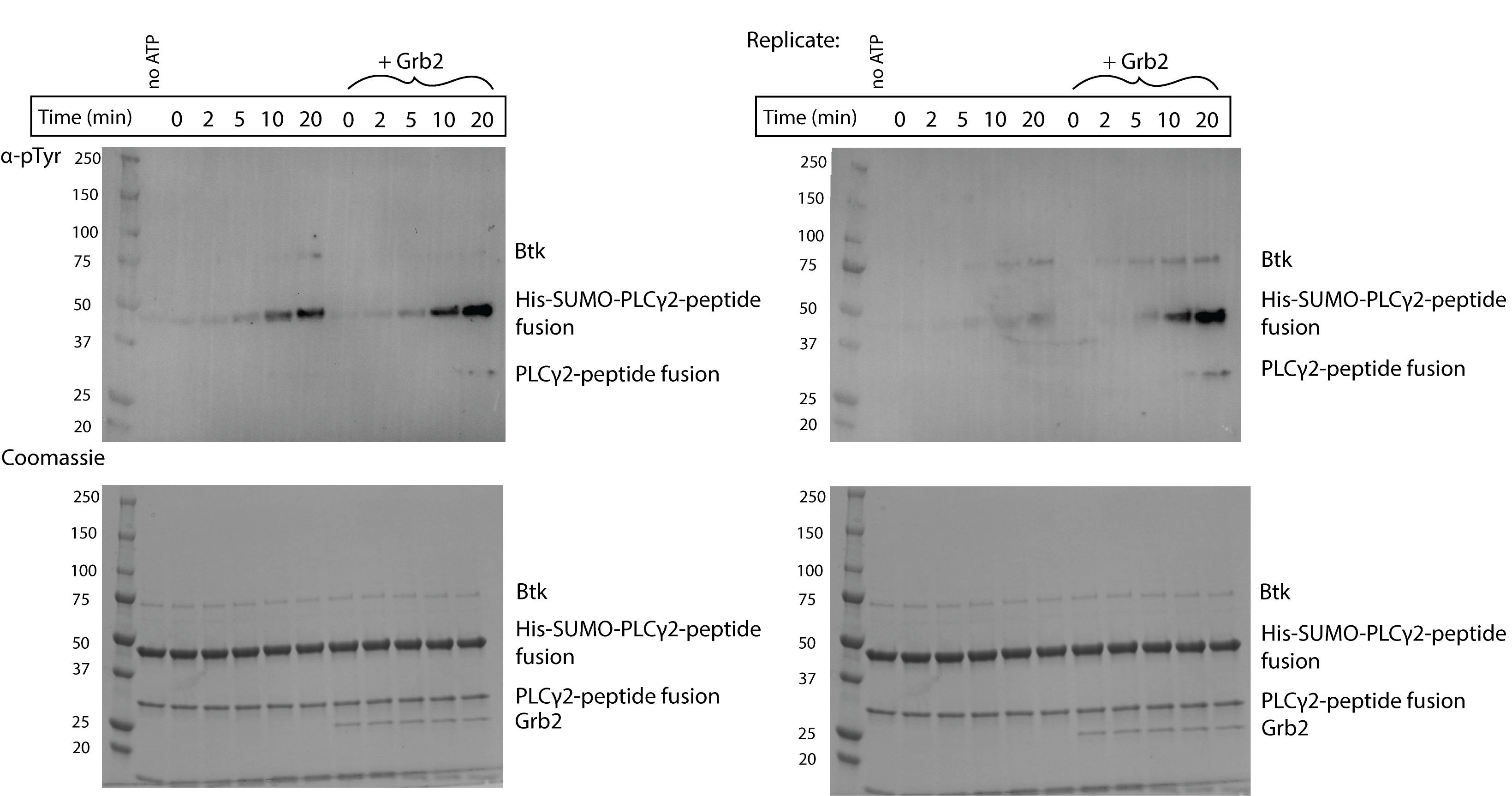

Supplement: Figure 3—source data 1. [file elife-82676-fig3-data1.zip › Figure 3 source data/Figure 3D-source data 5.png]

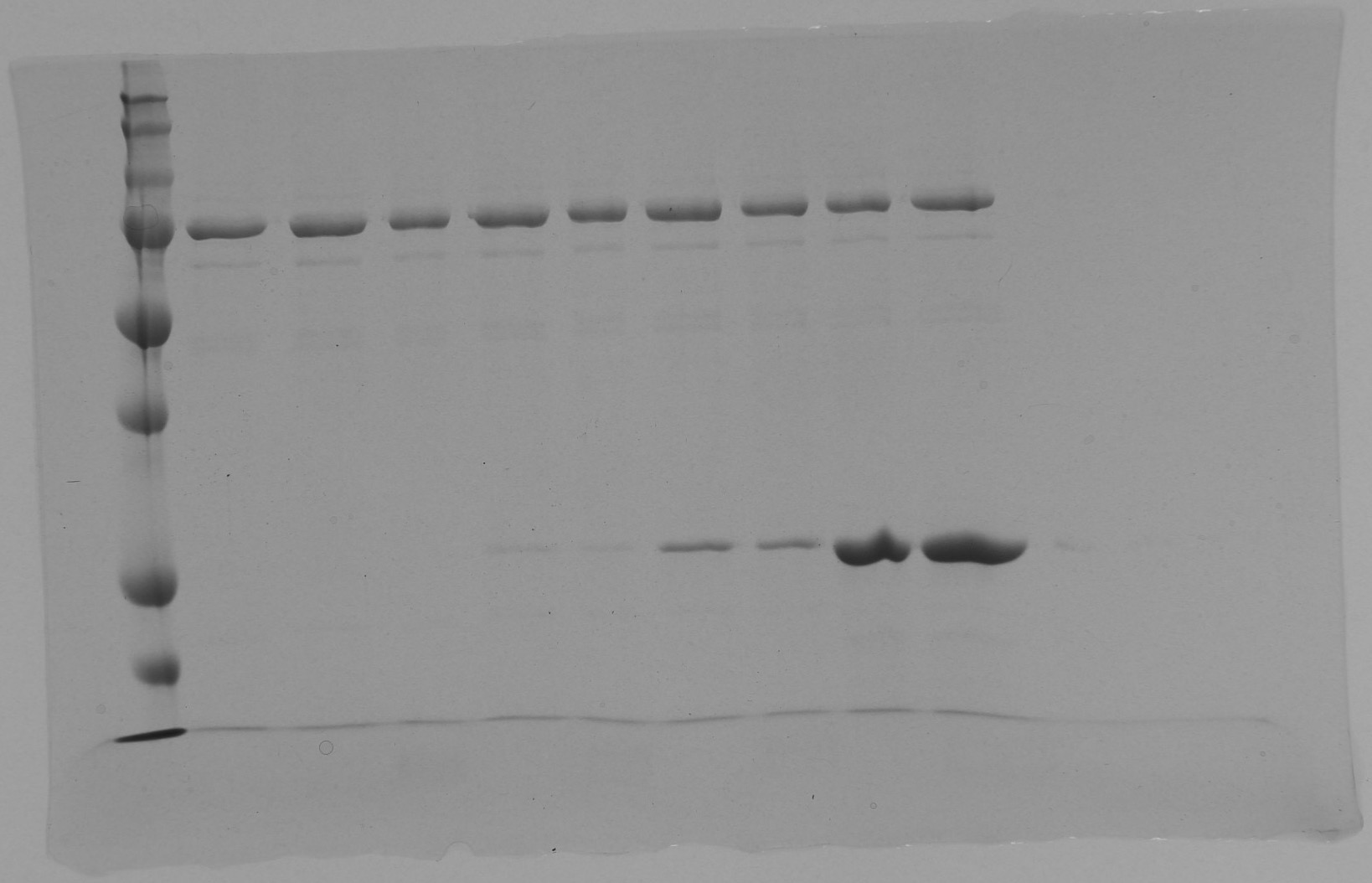

Supplement: Figure 3—source data 1. [file elife-82676-fig3-data1.zip › Figure 3 source data/Figure 3A-B-source data 4.jpg]

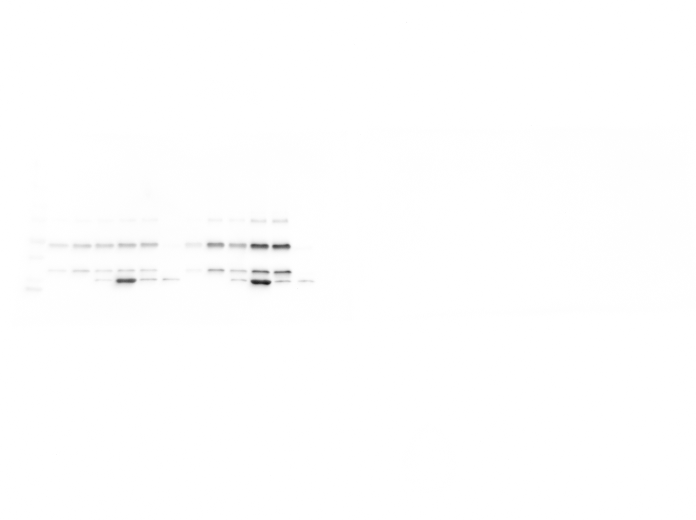

Supplement: Figure 3—source data 1. [file elife-82676-fig3-data1.zip › Figure 3 source data/Figure 3C-source data 1.tif]

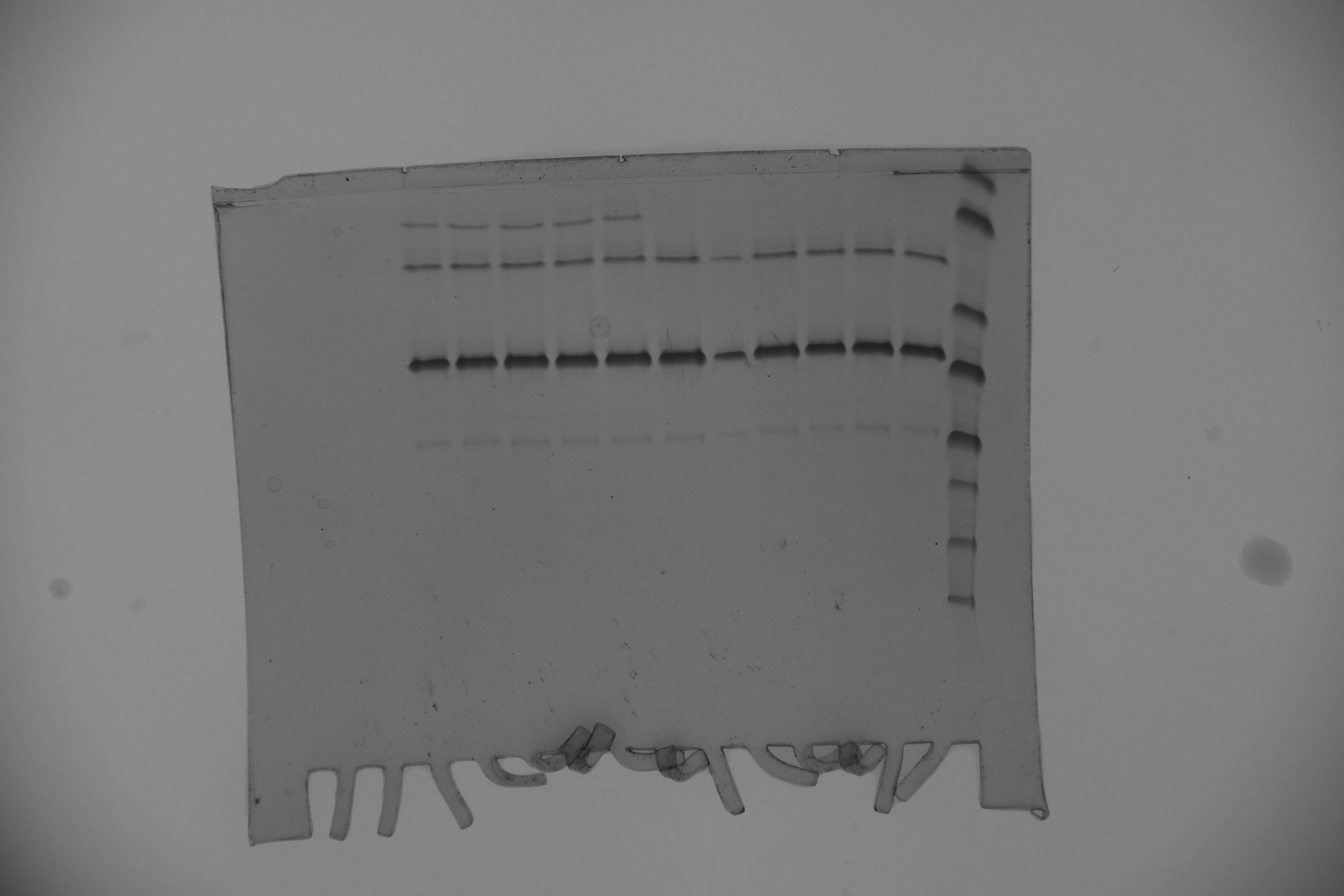

Supplement: Figure 3—source data 1. [file elife-82676-fig3-data1.zip › Figure 3 source data/Figure 3D-source data 4.JPG]

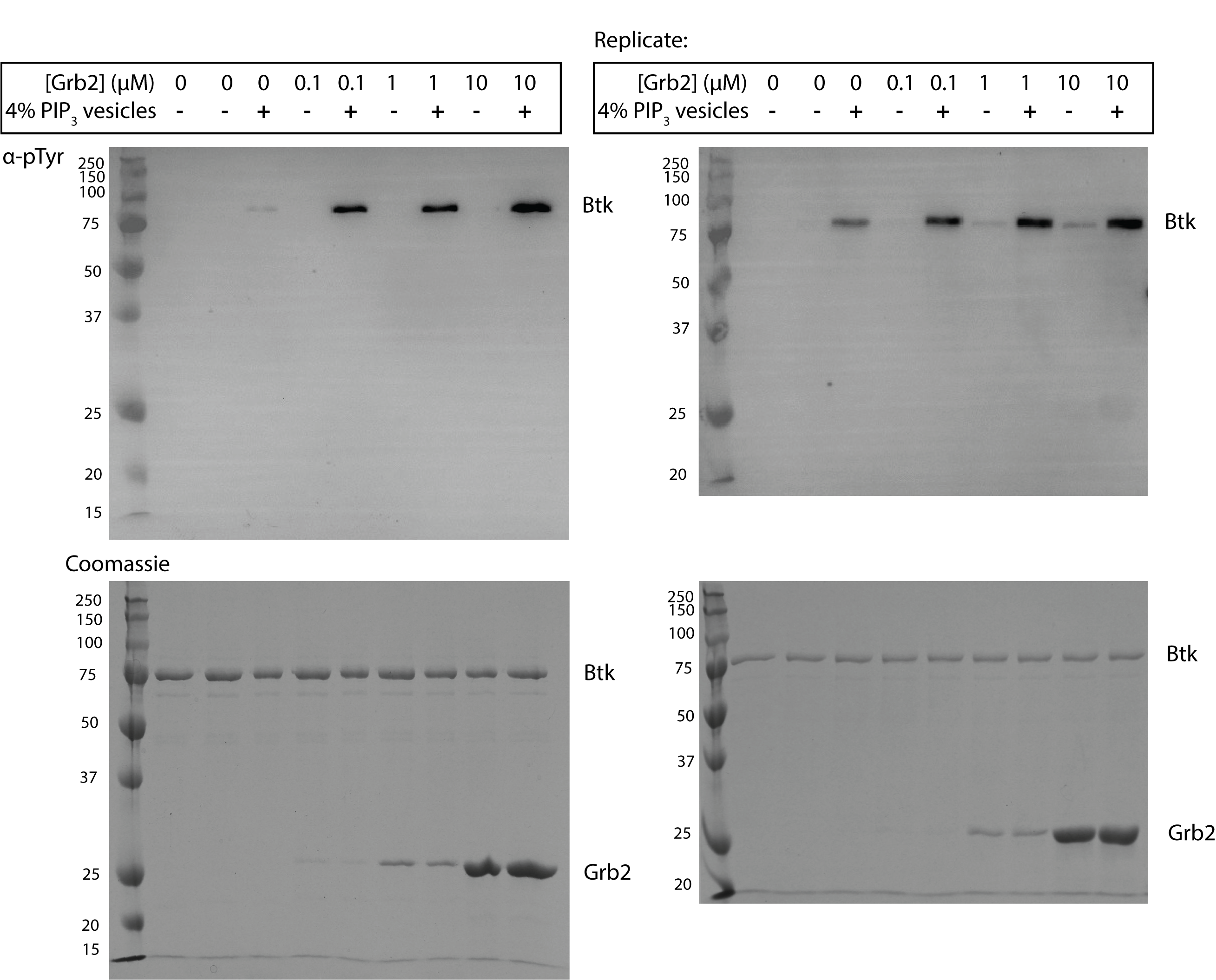

Supplement: Figure 3—source data 1. [file elife-82676-fig3-data1.zip › Figure 3 source data/Figure 3A-B-source data 5.png]

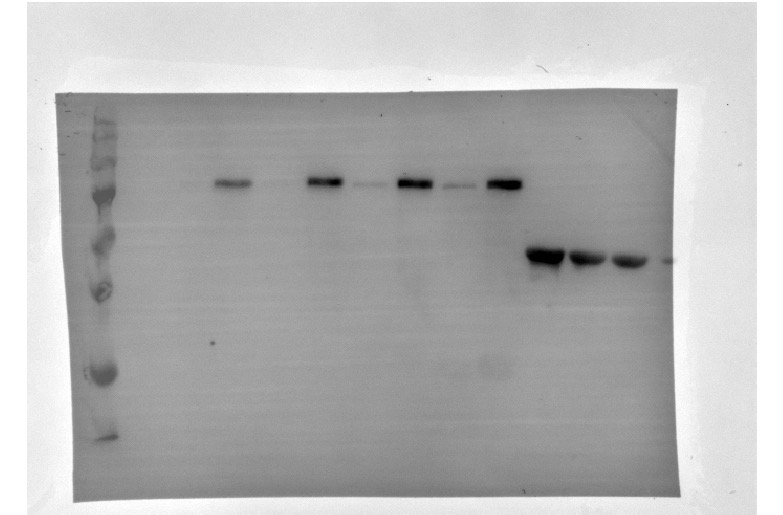

Supplement: Figure 3—source data 1. [file elife-82676-fig3-data1.zip › Figure 3 source data/Figure 3A-B-source data 1.jpg]

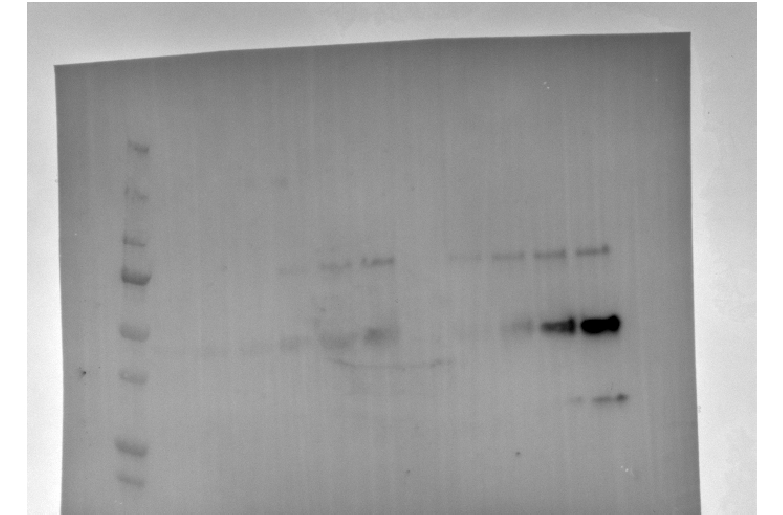

Supplement: Figure 3—source data 1. [file elife-82676-fig3-data1.zip › Figure 3 source data/Figure 3D-source data 1.jpg]

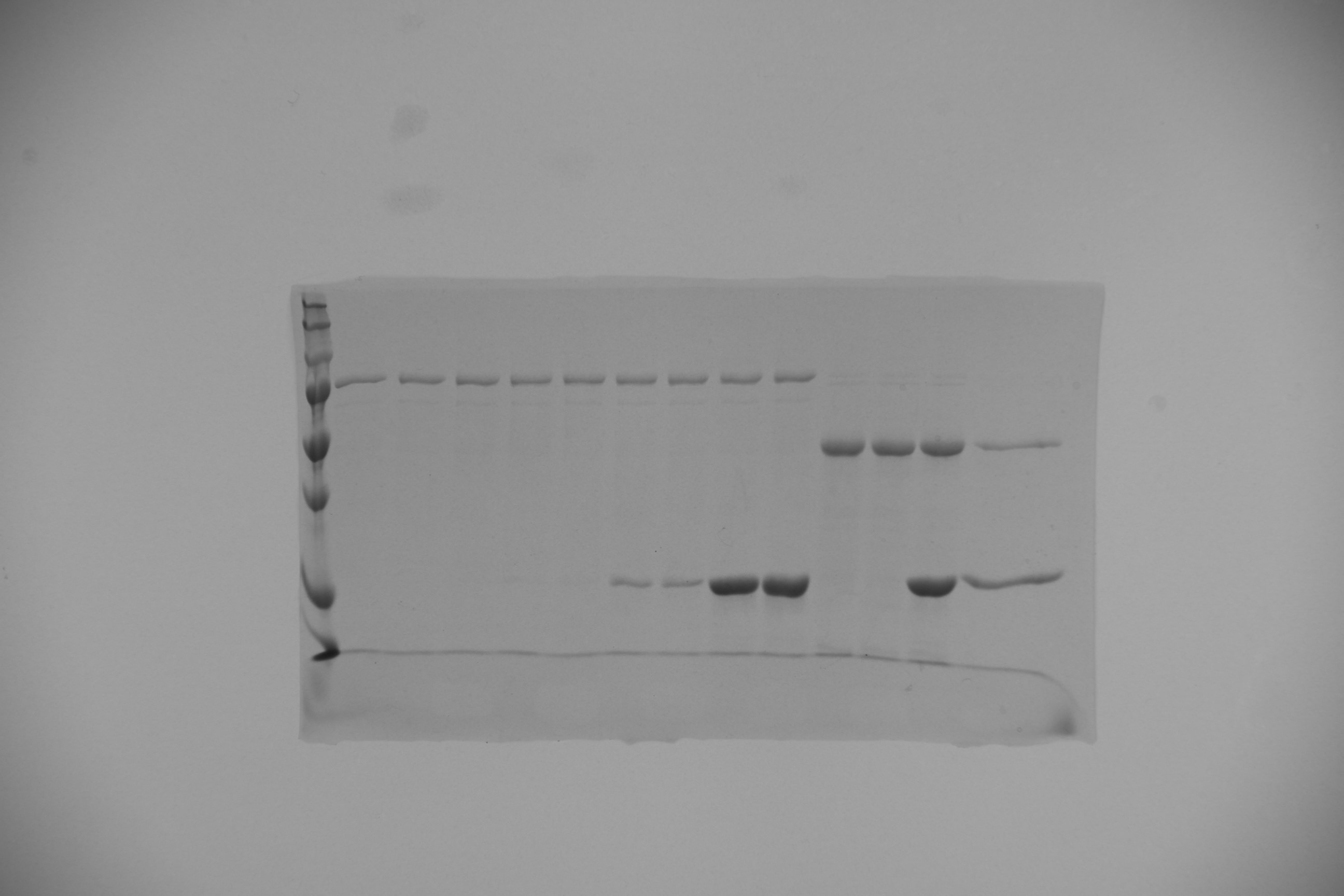

Supplement: Figure 3—source data 1. [file elife-82676-fig3-data1.zip › Figure 3 source data/Figure 3A-B-source data 2.jpg]

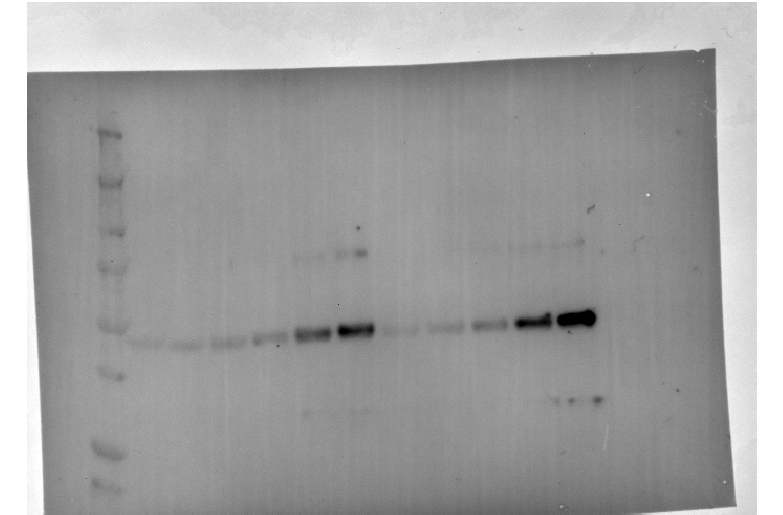

Supplement: Figure 3—source data 1. [file elife-82676-fig3-data1.zip › Figure 3 source data/Figure 3D-source data 3.jpg]

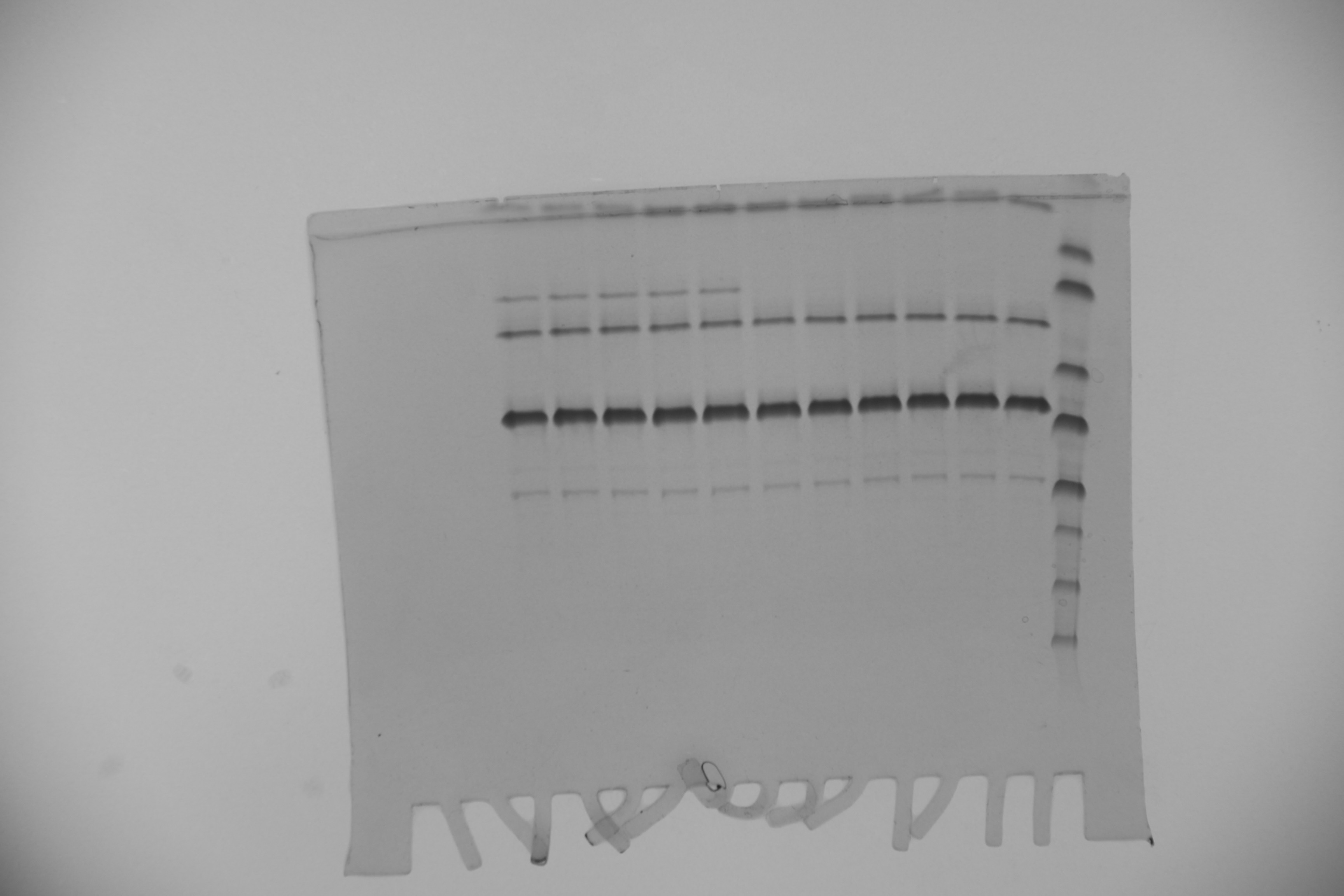

Supplement: Figure 3—source data 1. [file elife-82676-fig3-data1.zip › Figure 3 source data/Figure 3D-source data 2.jpg]

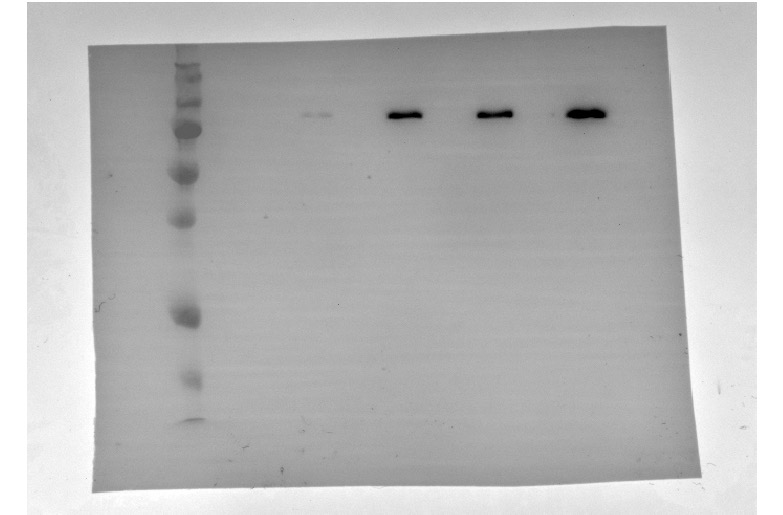

Supplement: Figure 3—source data 1. [file elife-82676-fig3-data1.zip › Figure 3 source data/Figure 3A-B-source data 3.jpg]

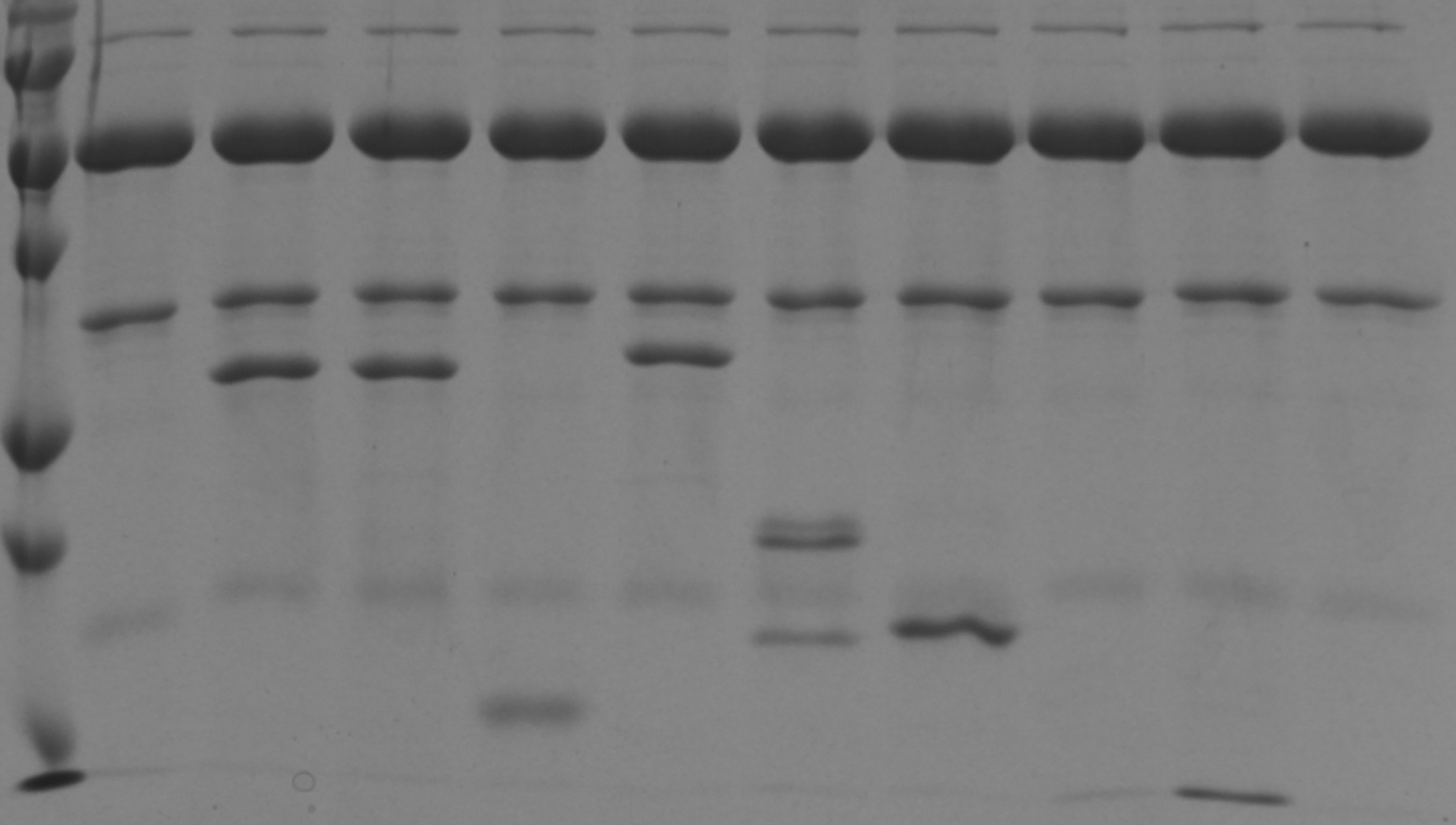

Supplement: Figure 4—source data 1. [file elife-82676-fig4-data1.zip › Figure 4 source data/Figure 4A-source data 2.jpeg]

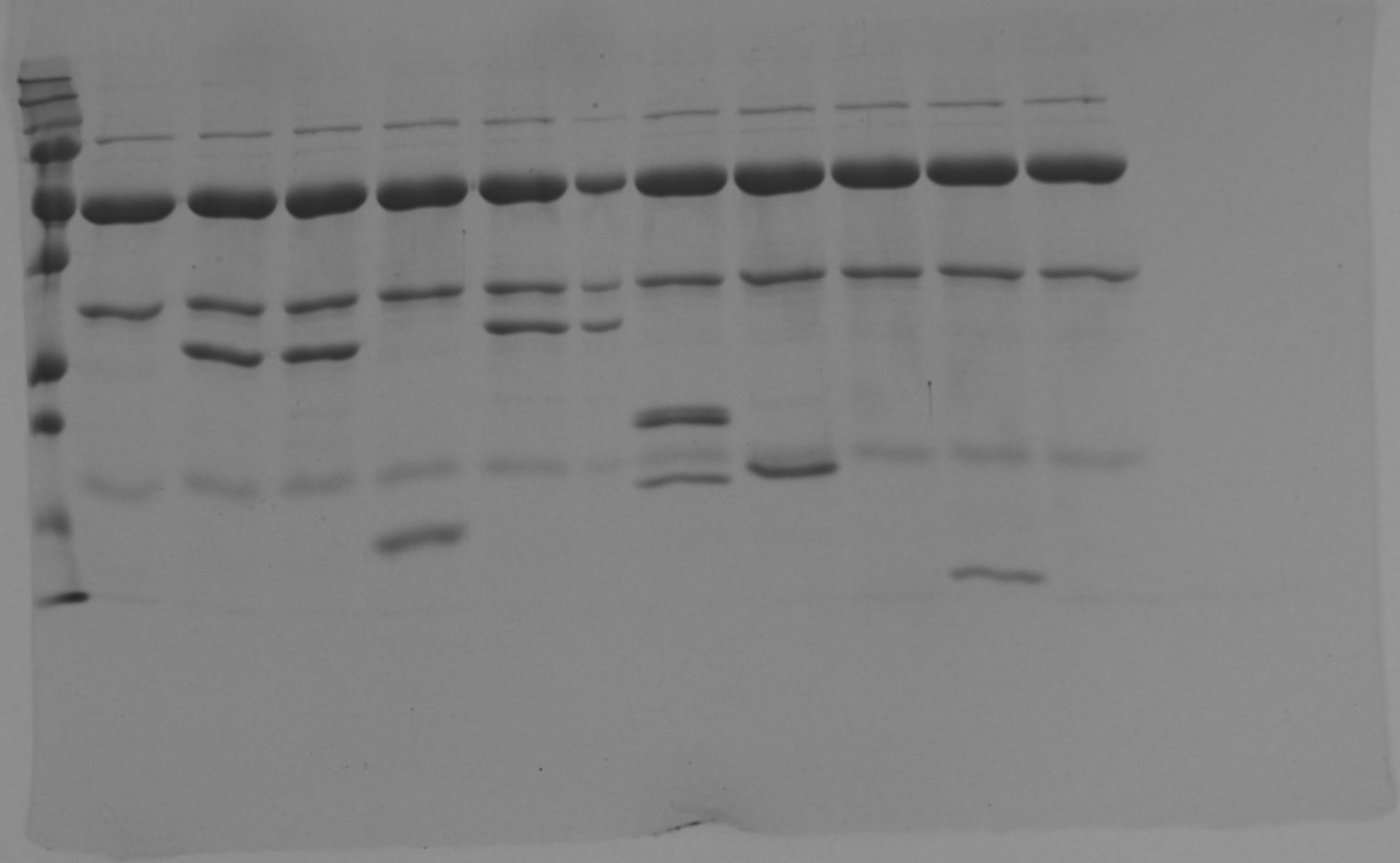

Supplement: Figure 4—source data 1. [file elife-82676-fig4-data1.zip › Figure 4 source data/Figure 4A-source data 4.jpg]

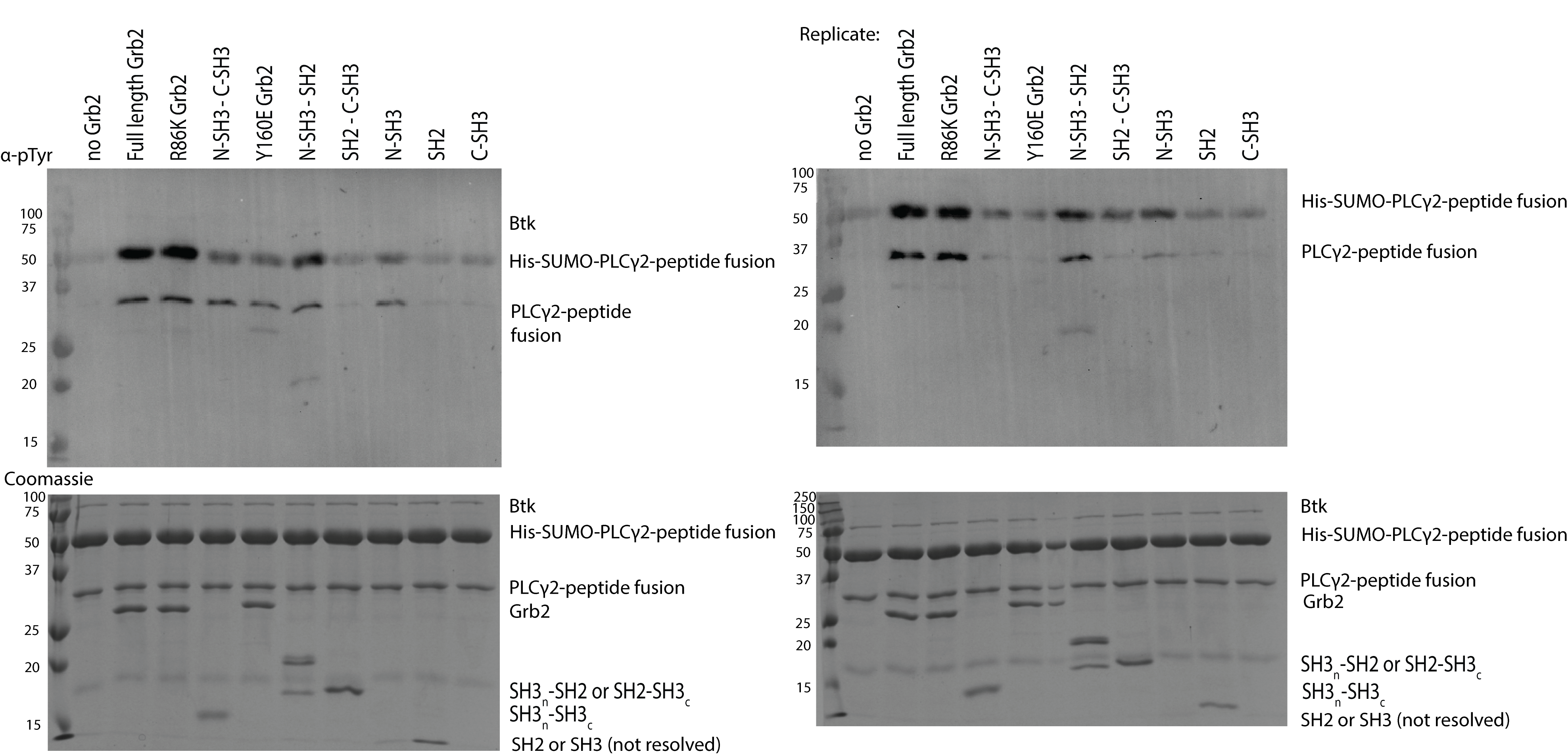

Supplement: Figure 4—source data 1. [file elife-82676-fig4-data1.zip › Figure 4 source data/Figure 4A-source data 5.png]

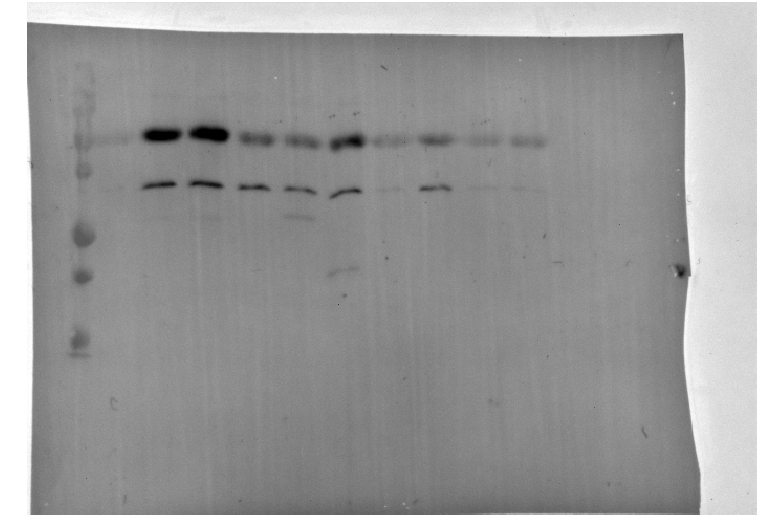

Supplement: Figure 4—source data 1. [file elife-82676-fig4-data1.zip › Figure 4 source data/Figure 4A-source data 1.jpg]

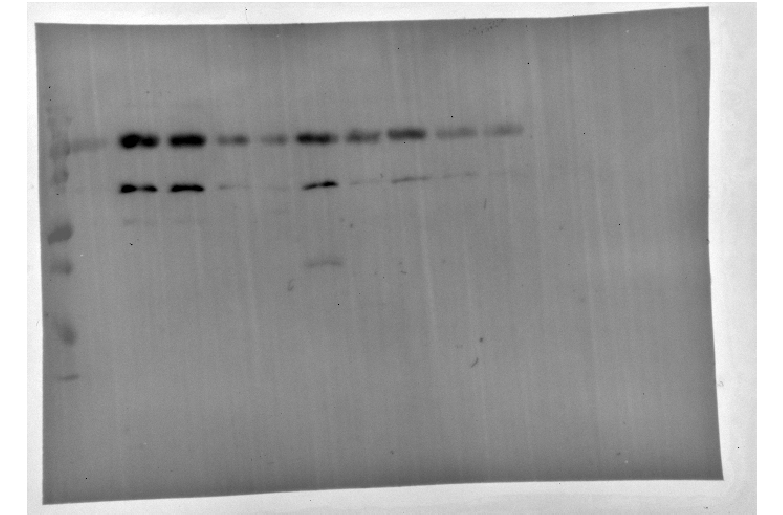

Supplement: Figure 4—source data 1. [file elife-82676-fig4-data1.zip › Figure 4 source data/Figure 4A-source data 3.jpg]
